# An optional surgical technique for obtaining lamellar donor grafts: a pilot study

**DOI:** 10.1186/s12886-022-02371-5

**Published:** 2022-03-25

**Authors:** Xin Liu, Chunyu Liu, Hui Lin, Yuting Shao, Li Zhang, Yanlong Bi

**Affiliations:** 1 Department of Ophthalmology, Tongji Hospital, School of Medicine, Tongji University, No. 389, Xincun Road, Putuo District, Shanghai, 200092 China; 2 Department of Ophthalmology, Guizhou Provincial People’s Hospital, Guiyang, Guizhou China; 3 Tongji Eye Institute, Tongji University School of Medicine, Shanghai, China

**Keywords:** Reversed manual dissection, Conventional manual dissection, Keratoplasty, Lamellar donor tissue, Surface quality, Thickness uniformity

## Abstract

**Background:**

To evaluate the surface quality and thickness uniformity of lamellar donor grafts using an optional surgical technique called reversed manual dissection (RMD) in porcine corneas.

**Methods:**

Twenty-four paired porcine corneas (48 eyes) were numbered 1 to 24 and divided into 6 groups. All left corneas were assigned to conventional manual dissection (CMD), and all right corneas were assigned to RMD. Each group contained 8 corneas. For Groups I, II, and III, 30, 50, and 70% of the entire corneal thickness was dissected using CMD. For groups IV, V, and VI, 70, 50, and 30% of the entire corneal thickness was dissected using RMD. The residual stromal thickness was examined by anterior segment optical coherence tomography (ASOCT) to assess the thickness uniformity and scanning electron microscopy (SEM) to assess the surface quality.

**Results:**

The thickness uniformity of the lamellar grafts between each paired group was not significantly different (*p >* 0.05). The qualitative surface roughness grading (QiSR) evaluated by masked observers through SEM was significantly higher in the RMD groups (*p* < 0.001). The quantitative surface roughness grading (QnSR) acquired from the Mountains software was significantly lower in the RMD groups (*p* < 0.001).

**Conclusions:**

RMD is an optional surgical technique for obtaining porcine lamellar grafts. The thickness uniformity of RMD is comparable to that of CMD, and a smoother surface with fewer ridges and roughness is achieved compared to CMD.

## Background

Anterior lamellar keratoplasty (ALK) has been widely used to treat keratoconus and many anterior stromal lesions. To a large extent, it preserves the structural integrity of the eyeball and reduces the postoperative steroid dosage [1]. The cornea is a transparent lenticular tissue that forms the anterior of the eye and provides two-thirds of its refractive power. To maintain its specific stiffness and optical properties, nearly 300 collagen lamellae are arranged and interwoven in multiple directions [2]. A poor interface quality and mismatched donor graft thickness negatively affect contrast sensitivity and visual performance. Thus, it is crucial to obtain a thickness-matched graft and the smoothest possible cutting plane.

Microkeratome and femtosecond laser-assisted corneal flaps during LASIK and lamellar grafts from donor corneas have been reported. However, in China, a large proportion of ophthalmologists still perform conventional manual dissection (CMD) in preparing donor tissue due to the lack of relevant equipment. During the procedure, the corneoscleral disc is mounted on an artificial anterior chamber (AAC) with the epithelium layer side up, and a diameter and depth-specific graft was dissected using a blade along the stromal fibers. However, the lamella could be inevitably damaged by mechanical traction. In clinical practice, we observed that the cutting plane of the donor lamellar cap is rougher than that of the stromal bed because there are many more ridges on the surface of the lamellar cap.

Based on this, we modified the lamellar dissecting method, called reversed manual dissection (RMD), in which the corneoscleral disc was mounted on an AAC with the endothelium layer side up. We followed up a group of patients and found a significant difference in surface quality between the two methods through corneal densitometry measured by the Pentacam AXL (Oculus, Germany), which could reflect the severity of the interlayer scars.

In our study, we compared the cutting plane quality and thickness uniformity of the porcine cornea between RMD and CMD for the first time.

## Methods

### Preparation of porcine eyeballs

All of the animals and experimental procedures were in line with the ARVO Statement for the Use of Animals in Ophthalmic and Vision Research and were conducted in accordance with ARRIVE guidelines. Twenty-four paired (48 eyes) of porcine eyeballs were enucleated immediately after slaughter, and the corneoscleral discs were preserved in the tissue storage medium (Optisol-GS, Bausch & Lomb, USA) at 4 °C for 24 h. The cornea was then mounted on a Barron’s artificial anterior chamber (Katena, Denville, USA), which was filled with air through the side port, to create a light reflection that allows the surgeon to estimate the depth during dissection and maintain the pressure at 70 mmHg. The corneal thickness was measured by anterior segment optical coherence tomography (ASOCT; Carl Zeiss Meditec, Germany) using a global pachymetry model after removing the epithelium using a cellulose sponge. We calculated the average corneal thickness in the central 5 mm range based on the global pachymetry result.

### Surgical technique

Twenty-four paired (48 eyes) porcine corneoscleral discs were numbered 1 L to 24 L and 1 R to 24 R, where 1 L means cornea from the left eye of the porcine numbered 1 and so on and 1 R means cornea from the right eye of the porcine numbered 1 and so on. All corneas were divided into 6 groups according to the cutting depth (Table 1). For Groups I, II, and III, 30, 50, and 70% of the entire corneal thickness was dissected using CMD. For groups IV, V, and VI, 70, 50, and 30% of the entire corneal thickness was dissected using RMD.

**Table 1:** Grouping and graft information

Group	N	Dissection method	Punch diameter (mm)	Average predissected CCT (μm)	Target cutting depth setting(%)	Average residual stromal thickness (μm)	Average actual cutting depth (μm)	Average actual donor graft thickness (%)
I	8	CMD	8.25	1096.17	30	787.59	308.58	27.96
II	8	CMD	8.25	1087.87	50	521.96	521.96	47.98
III	8	CMD	8.25	1101.68	70	353.60	748.08	67.90
IV	8	RMD	8.25	1103.42	70	323.49	779.93	29.36
V	8	RMD	8.25	1089.57	50	554.44	535.14	49.12
VI	8	RMD	8.25	1061.66	30	729.04	332.62	68.63

*CCT* Average central 5 mm corneal thickness measured by AS-OCT

**Fig. 1:**
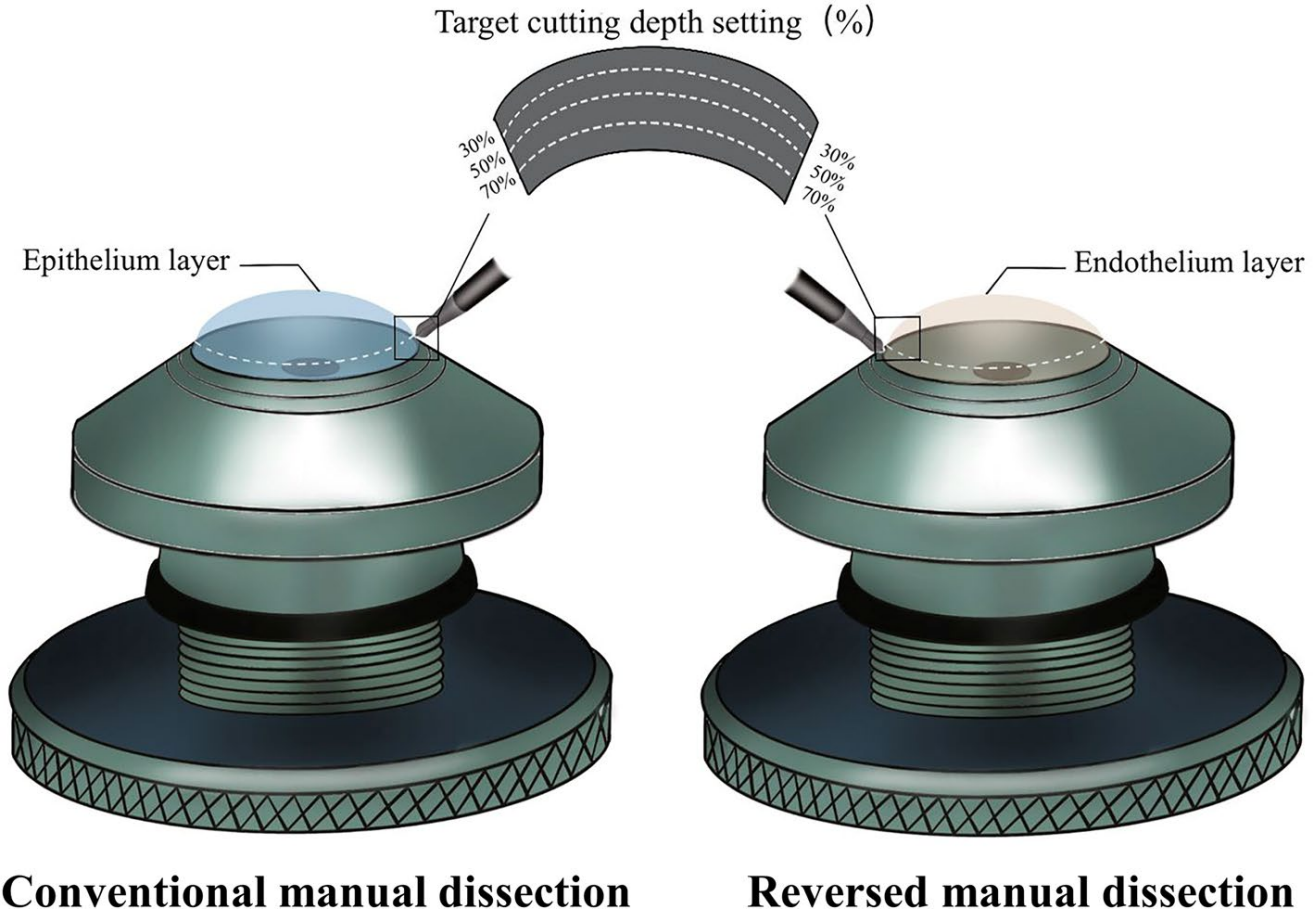
Schematic diagram of the conventional manual dissection and the reversed manual dissection

For groups I, II, and III, the left corneoscleral discs were respectively mounted on AAC with the epithelium layer side up. Taking the corneal apex as the center, lamellar with a diameter of 8.25 mm and cutting depths of 30, 50, and 70% of the average central 5 mm corneal thickness were made under the assistance of a vacuum trephine (Katena products, Inc., USA) with a blade along the stromal fibers. For groups IV, V, and VI, the right corneoscleral discs were obtained using a modified method, named RMD. In this method, the corneoscleral disc was mounted on AAC with the endothelium layer side up. After dissecting the posterior lamella with a diameter of 8.25 mm and a cutting depth of 70, 50, and 30% of the average central 5 mm corneal thickness, the corneoscleral disc was dismounted and punched to the same size as the CMD groups. The schematic diagram was shown in Fig. 1.

### Scanning Electron microscopy (SEM)

Dissected lamellar donor grafts were immediately immersed in 2.5% glutaraldehyde and fixed at 4 °C overnight for further processing. Lamellar grafts were immediately washed with phosphate-buffered saline (PBS) 3 times and then were immersed in 1% osmium acid solution at 4 °C for 2 h. Specimens were washed with PBS, then dehydrated in a series of gradient ethanol baths, and then dried to the critical point. The grafts were then sputter-coated, and examined with SEM (Hitachi S-SU8010).

### Qualitative surface roughness grading (QiSR)

The lamellar grafts were scored based on the electron micrograph by three masked observers using a previously described scoring method [3, 4], as shown in Table 2.

**Table 2:** Criteria for evaluating surface characteristics

	Criterion and Magnification	Appearance	Scores
A	Surface relief ×100	Very smooth	4
		Smooth	3
		Rough	2
		Very rough	1
B	Regularity of surface structure ×300	Completely regular	4
		Almost Regular	3
		Partially regular	2
		Not regular	1
C	Portion of surface irregular ×300	< 10% of cut surface	4
		11–25% of cut surface	3
		26–50% of cut surface	2
		> 50% of cut surface	1
D	Position of the irregular area ×300	No irregularities	4
		Peripheral only	3
		Large region	2
		All over	1

### Quantitative surface roughness grading (QnSR)

The central × 100 SEM image of the stromal surface was analyzed with the Mountains software (MountainsSEM v.9; Digital Surf, Besançon, France). The software calculated the height of surfaces from the fractal dimensions to assess the surface roughness and was designed for evaluating surface roughness in the SEM images according to the ISO 25178 standard [5–7]. The higher the score, the rougher the surface.

### Graft thickness uniformity evaluation

The ASOCT self-contained model ‘Global Pachymetry’ was used to measure the corneoscleral thickness. A black dot was marked on the edge of the AAC. Pachymetry scans were performed on the grafts after 4 procedural steps: removal of the epithelium, mounted on the AAC, always keeping the black dot at 12 o’clock, and scanning in the ‘Global Pachymetry’ model. After dissection, the residual corneoscleral discs were scanned again to calculate the graft thickness.

### Statistical analysis

All analyses were performed in SPSS version 20.0 (SPSS, Chicago, USA). The Kolmogorow-Smirnow test was used to test the normality of the difference, and the independent sample t-test or Mann-Whitney U test were used to assess the differences between groups. *p* < 0.05 was considered to be statistically significant. Spearman’s rank correlation coefficient was calculated to assess the reproducibility of the two roughness assessment methods (QiSR and QnSR).

## Results

All corneoscleral discs were examined under a slit lamp before dissection, and no obvious abnormality was found. Under microscope examination, the graft shape in the CMD groups was slightly deformed due to the pulling of forceps during the dissection. However, there was almost no deformation in the RMD groups because of the uniform force during dissection and trephination. At the same time, the graft surfaces were much smoother and more regular in the RMD groups.

### Graft thickness uniformity measured by ASOCT

**Fig. 2:**
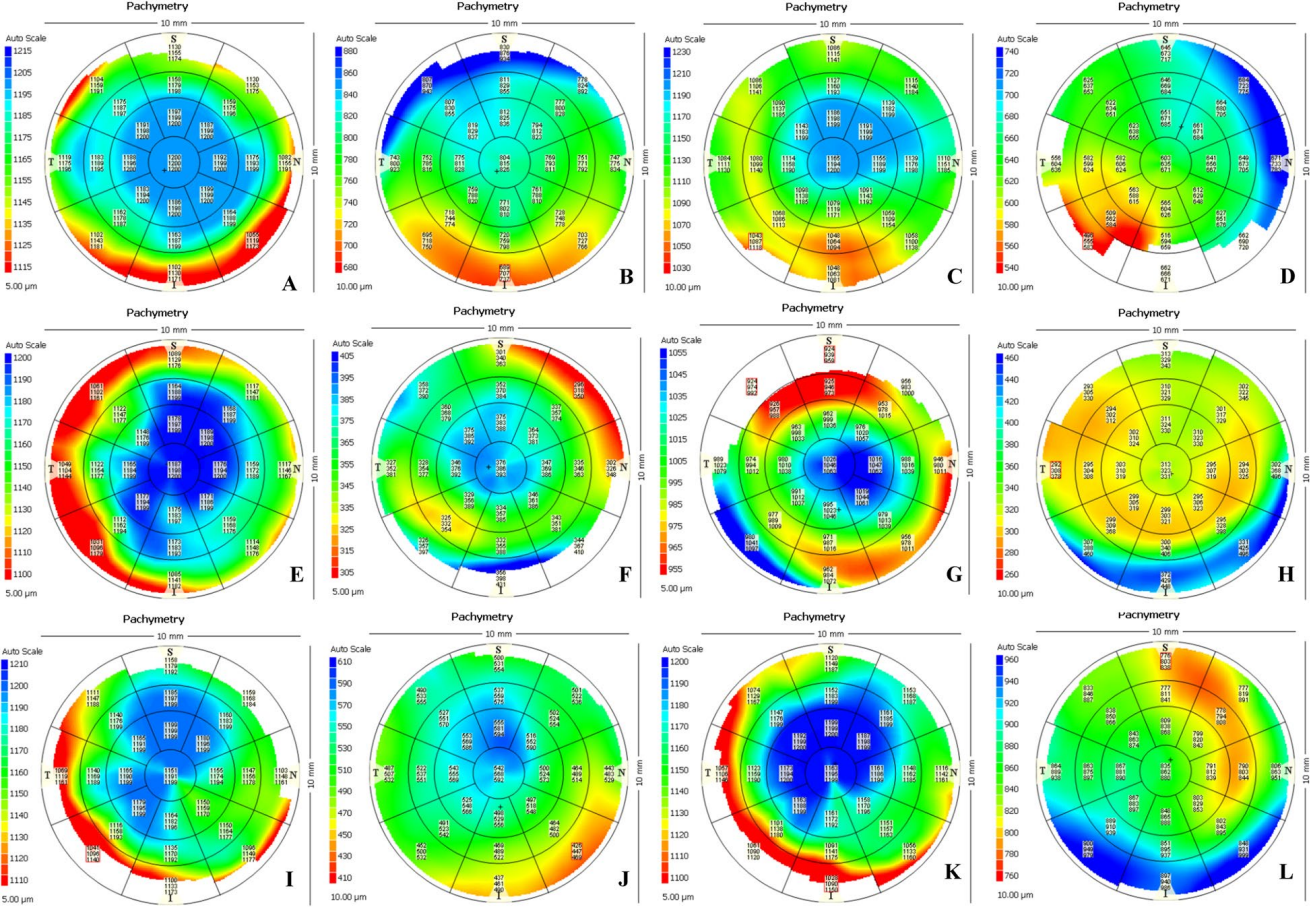
Representative pachymetric images measured by ASOCT. **A** Predissected image of 30% of the corneal thickness. **B** Postdissected image of 30% of the corneal thickness using CMD. **C** Predissected image of 50% of the corneal thickness. **D** Postdissected image of 50% of the corneal thickness using CMD. **E** Predissected image of 70% of the corneal thickness. **F** Postdissected image of 70% of the corneal thickness using CMD. **G** Predissected image of 70% of the corneal thickness. **H** Postdissected image of 70% of the corneal thickness using RMD. **I** Predissected image of 50% of the corneal thickness. **J** Postdissected image of 50% of the corneal thickness using RMD. **K** Predissected image of 30% of the corneal thickness. **L** Postdissected image of 30% of the corneal thickness using RMD

As shown in Table 1, the mean central 5 mm corneal thickness of the predissection corneas among the 3 paired groups (Group I versus IV, Group II versus V, and Group III versus VI) was not significantly different (*p* = 0.848, *p* = 0.960, and *p* = 0.271). The graft thickness uniformity was not significantly different after dissection among the different cutting depth groups (*p* = 0.523, *p* = 0.163, and *p* = 0.498), which were all similar to the targeting cutting depth. Representative pachymetric images are shown in Fig. 2.

### Surface quality evaluated by SEM

**Fig. 3:**
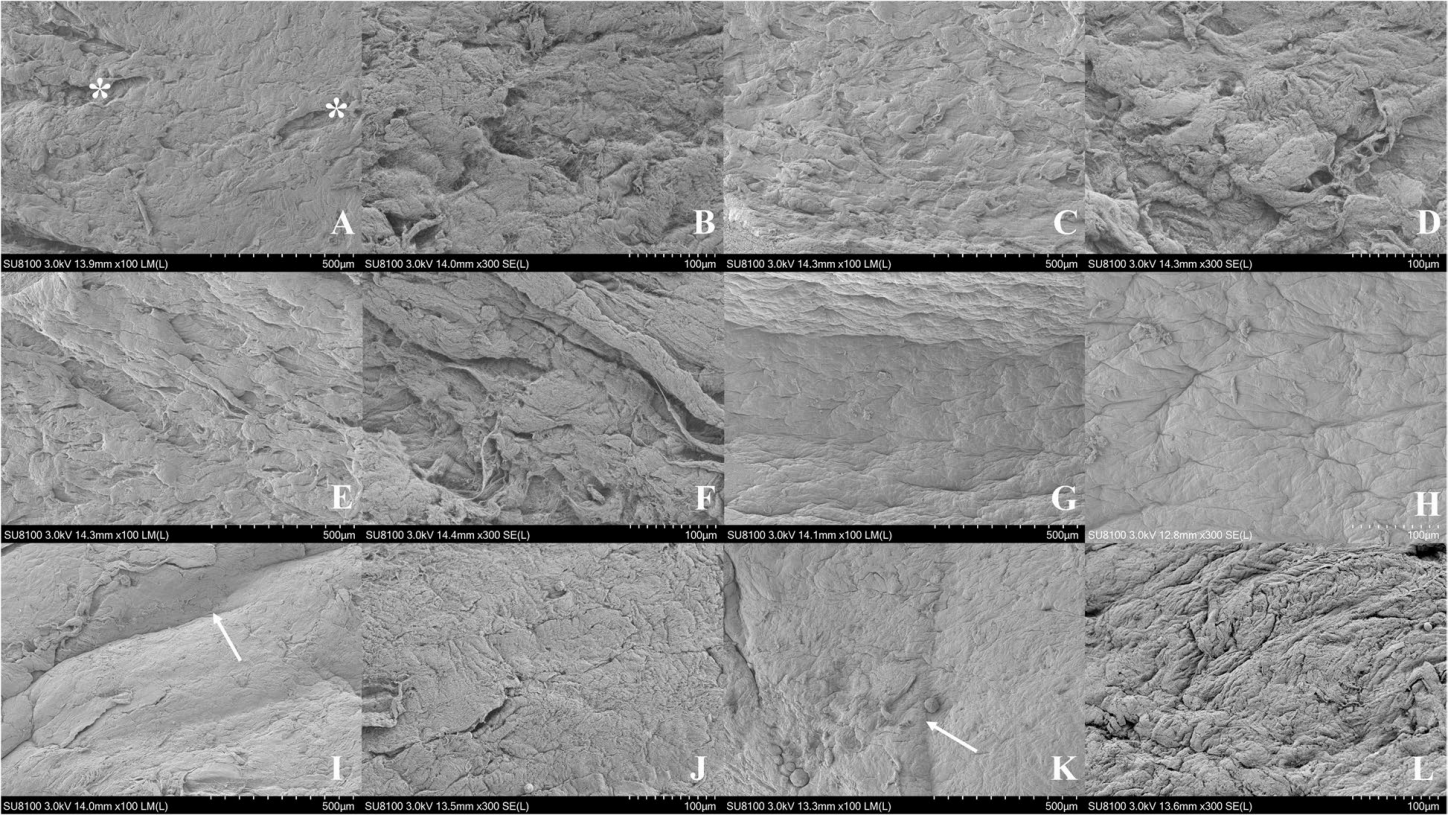
Representative SEM images of different dissecting depths through CMD and RMD. **A** 30% dissected graft using CMD at × 100 magnifications. CMD group showed more cutting scratchs (asterisk). **B** 30% dissected graft using CMD at × 300 magnifications. **C** 50% dissected graft using CMD at × 100 magnifications. **D** 50% dissected graft using CMD at × 300 magnifications. **E** 70% dissected graft using CMD at × 100 magnifications. **F** 70% dissected graft using CMD at × 300 magnifications. **G** 70% dissected graft using RMD at × 100 magnifications. **H** 70% dissected graft using RMD at × 300 magnifications. **I** 50% dissected graft using RMD at × 100 magnifications. There were long and deep grooves (arrow) caused by the ethanol-dependent dehydration process necessary for SEM. **J** 50% dissected graft using RMD at × 300 magnifications. **K** 30% dissected graft using RMD at × 100 magnifications. **L** 30% dissected graft using RMD at × 300 magnifications

The microstructure of the donor grafts was observed under SEM at × 100, × 300 magnifications. There were long and deep grooves caused by the ethanol-dependent dehydration process necessary for SEM in both groups. The CMD groups showed scattered iatrogenic fractured collagens caused by blades, while this hardly existed in the RMD groups. Meanwhile, the CMD groups presented more linear uplifts, curls, and tissue bridges on the surface, making them much rougher than the RMD groups. Figure 3 shows representative images of the grafts.

### Stromal roughness grading

The mean QiSR of the CMD and RMD groups was 8.36 ± 1.25 (range 6–12) and 11.88 ± 1.10 (range 9–14), respectively (*p* = 0.000). The mean QiSR in Group I and Group IV was 9.25 ± 1.94 (range 7–12) and 12.13 ± 2.73 (range 9–14), respectively (*p* = 0.000). The mean QiSR in Group II and Group V was 8.04 ± 1.07 (range 6–10) and 11.97 ± 1.94 (range 10–14), respectively (*p* = 0.000). The mean QiSR in Group III and Group VI was 7.79 ± 1.69 (range 6–10) and 11.54 ± 0.64 (range 10–13), respectively (*p* = 0.000) (Table 3).

**Table 3:** Score results of the surface quality evaluated by SEM

Group	N	Qualitative surface roughness grading (QlSR)						Quantitative surface roughness grading (QnSR)	
		Surface Relief	Regularity of Surface	Portion of Surface Irregular	Position of the irregular area	Scores	SD	Scores	SD
I	8	2.58	2.08	2.17	2.42	9.25	1.94	24.32	3.99
II	8	2.33	1.96	1.79	1.96	8.04	1.07	33.62	6.83
III	8	2.12	1.92	1.58	2.17	7.79	1.69	37.84	5.33
IV	8	3.46	2.88	3.00	2.79	12.13	2.73	10.37	1.61
V	8	3.37	2.83	3.00	2.76	11.97	1.94	13.98	1.58
VI	8	3.17	2.92	2.75	2.71	11.54	0.64	14.20	1.60

The mean QnSR of the CMD and RMD groups was 31.92 ± 7.81 (range 18.53–45.92) and 12.85 ± 2.36 (range 8.35–16.23). The mean QnSR in Group I and Group IV was 24.32 ± 3.99 (range 18.53–29.94) and 10.37 ± 1.61 (range 8.35–12.66), respectively (*p* = 0.000). The mean QnSR in Group II and Group V was 33.62 ± 6.83 (range 21.80–43.14) and 13.98 ± 1.58 (range 11.37–16.23), respectively (*p* = 0.000). The mean QnSR in Group III and Group VI was 37.84 ± 5.33 (range 30.73–45.92) and 14.20 ± 1.60 (range 11.62–16.20), respectively.

**Fig. 4:**
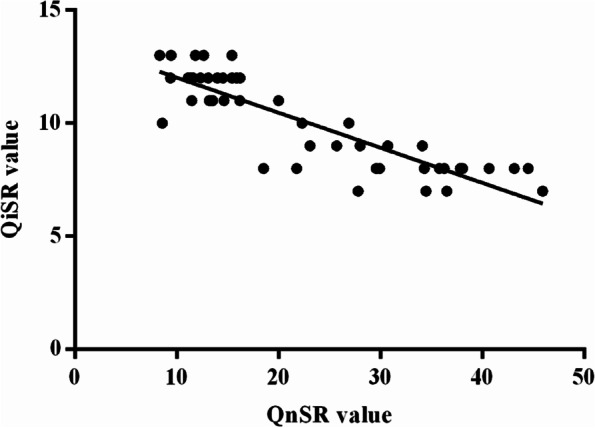
Comparison of the two methods used to evaluate the surface quality. Qualitative surface roughness grading (QiSR) was performed by masked observers and quantitative surface roughness grading (QnSR) was performed using the Mountains software (MountainsSEM v.9; Digital Surf, Besançon, France). There was a correlation of these two methods with Spearman’s rank correlation coefficient (R_s_ = − 0.835)

Spearman’s rank correlation coefficient was calculated to assess the reproducibility of the two roughness assessment methods (QiSR and QnSR). QiSR was significantly negatively correlated with QnSR (R_s_ = − 0.835) (Fig. 4).

## Discussion

ALK is a partial keratoplasty used when the stroma is damaged and the endothelium is intact. It accounts for approximately 40% of all keratoplasties. This partial keratoplasty is beneficial compared to penetrating keratoplasty because it reduces the corneal endothelial immune reaction and results in better outcomes [8, 9]. There are various forms of ALK surgery being used today to adapt to a wide range of corneal stromal diseases. These include manual ALK surgery, descemetic-deep anterior lamellar keratoplasty (DALK), predescemetic-DALK, microkeratome-assisted ALK and femtosecond laser-assisted ALK [10]. Compared with manual dissection, microkeratome/femtosecond laser-assisted ALK provides greater precision and reproducibility during lamellar dissection [11–13]. However, not all corneal transplantation centers have these devices, so manual dissection is still the predominant surgical method being used in China.

Traditional manual dissection techniques result in suboptimal visual qualities due to interface haze and scarring [10]. A smooth interface is crucial for good visual outcomes, which has been demonstrated by the advantages of DALK over conventional lamellar keratoplasty [14, 15] and big-bubble dissection over manual dissection in DALK [16, 17].

In this paper, we indicated an optional surgical technique in preparing donor corneal grafts, called RMD. To compare the modified and conventional surgical techniques more comprehensively, we evaluated these two techniques from the aspects of surface quality and thickness uniformity. Ultrastructures of different dissecting depths (30, 50, and 70% of stromal thickness) were compared in our study. Our results showed that both the RMD and CMD methods provided equivalent predictability and reproducibility in corneal thickness uniformity. RMD had more advantages in graft cutting plane smoothness. We speculated that the reasons for these results are as follows: First, in clinical practice, it is much easier to observe the stromal bed than the lamellar cap under a microscope, so surgeons can more readily guarantee the surface quality of the stromal bed. In addition, stromal beds mounted on AACs always maintain uniform traction. During the conventional dissecting process, to better expose the dissecting plane, surgeons always use tweezers to pull the graft mechanically, but excessive clamping will not only cause iatrogenic damage to graft, but also lead to graft deformation. However, reversed dissection could avoid these drawbacks.

Tissue bridges are defined as residual fibers on the surface after laser cutting and are the main entity of irregularities [4]. Zhang et al. have stated that ridges on the surface were significantly correlated with dissecting depth, and were more strongly correlated with the percentage of dissecting depth. They also stated that the deeper the dissection was, the more ridges there were. A depth setting of 31% stromal thickness or shallower might produce adequate surface quality for femtosecond laser-assisted ALK [18]. This is consistent with our results. As the cutting depth deepened, SEM images showed more ridges and tissue bridges. Previous studies have reported that the anterior lamellae collagen has a spring-shaped arrangement, whereas posterior lamellae collagen is arranged in an orthogonal direction [19]. Hence, we speculate that a parallel space between fibrils minimizes fractured fibrils during dissection.

The limitation of our study is that we used porcine corneas, not the human corneas. It is essential to consider the biological differences between porcine and human corneas. Many researches have stated that porcine corneas have different characteristics to human corneas in size, thickness, curvatures and some biomechanical properties [20, 21]. However, the shortage of donors prevented us from using human corneas. The reasons why we chose porcine corneas were as follows: 1. Previous studies have shown that fresh porcine cornea and decellularized porcine cornea (DPC) presented a promising result in the in vivo keratoplasty for non-human primates (NHP) and human [21, 22]; For ALK with small diameter, porcine grafts have been reported to survive for about 90–180 days even without immunosuppression [23–25]. For DALK cases used anti-CD40 Ab, porcine grafts survived more than 180 days [26]. DPC has been approved to be used for ALK by the Chinese National Institutes for Food and Drug Control [27]. And the graft showed long-term survival both in NHP and human studies [28, 29]. Therefore, our study is still worthy because this outcome can provide some of the basic evidence to possibly use for human graft. 2. In the past, many other studies also used porcine corneas to evaluate the stromal surface [7, 30, 31]. 3. Porcine cornea is easy to obtain and cheap. We believe that our findings have increased our understanding of the different results that may be obtained after different dissecting methods and may provide an alternative method for obtaining smoother surfaces.

## Conclusions

This study suggests that RMD is a comparable surgical technique to CMD for obtaining porcine lamellar graft. Our results show that both the RMD and CMD methods provide considerable predictability and reproducibility in thickness uniformity; however, RMD has more advantages in obtaining a smoother dissecting surface in porcine corneas.

## Data Availability

All data used and analyzed in this study are available upon request from the first author: Xin Liu.

## Abbreviations

ALK: Anterior lamellar keratoplasty
CMD: Conventional manual dissection
AAC: Artificial anterior chamber
RMD: Reversed manual dissection
ASOCT: Anterior segment optical coherence tomography
SEM: Scanning electron microscopy
PBS: Phosphate buffer saline
DALK: Deep anterior lamellar keratoplasty
QlSR: Qualitative surface roughness grading
QnSR: Quantitative surface roughness grading
DPC: Decellularized porcine cornea
NHP: Non-human primates
